# Amoxicillin-Induced Hemolytic Uremic Syndrome and Kidney Injury: A Case Report

**DOI:** 10.7759/cureus.77082

**Published:** 2025-01-07

**Authors:** Ashok Abraham Varughese, Madeeha Subhan Waleed, Radhika Pathalapti

**Affiliations:** 1 Internal Medicine, Lower Bucks Hospital, Bristol, USA; 2 Nephrology, Lower Bucks Hospital, Bristol, USA

**Keywords:** amoxcillin, clinical hematology, drug induced aki, general nephrology, hemolytic uremic syndrome (hus), internal medicine

## Abstract

Amoxicillin (AMX) is a commonly used antibiotic for treating infections and as a prophylactic antimicrobial agent, appreciated for its efficacy and favorable pharmacokinetics. Drug-induced acute kidney injury (AKI) significantly increases morbidity and mortality. Hemolytic uremic syndrome (HUS) is classified under thrombotic microangiopathies (TMAs), which are characterized by hemolysis, low platelet counts, thrombus formation in small vessels, and end-organ damage. While AMX-induced HUS has not been previously reported, AMX can cause AKI through mechanisms such as acute interstitial nephritis and AMX-induced crystal nephropathy (AICN), with AICN being more common. We present the case of a 56-year-old woman who developed AMX-induced HUS and AKI following AMX administration for a tooth infection. A kidney biopsy revealed distinctive glomerular damage, consistent with acute tubular injury and focal segmental glomerulonephritis. The diagnosis of drug-induced kidney injury with concurrent TMA was confirmed. AMX was discontinued, and the patient received plasmapheresis and hemodialysis. Typically, renal impairment is reversible once the offending agent is withdrawn. Physicians should be aware of the potential for AMX-induced HUS. Comprehensive medical history, physical examination, and prompt therapeutic intervention are crucial for effective treatment and improved patient outcomes.

## Introduction

Amoxicillin (AMX), either alone or in combination with clavulanate, is widely used for treating infections and as a prophylactic antimicrobial due to its efficacy and favorable pharmacokinetics. Major adverse effects of AMX include hypersensitivity reactions and drug-induced crystal nephropathy (AICN). Drug-induced acute kidney injury (AKI) remains a significant cause of morbidity and mortality [1,2]. Hemolytic uremic syndrome (HUS), a type of thrombotic microangiopathy (TMA), is characterized by hemolysis, low platelet counts, thrombus formation in small vessels, and end-organ damage [3]. Although AMX-induced HUS has not been reported, AMX can cause AKI through mechanisms such as acute interstitial nephritis (AIN) and AICN, with AICN being more common [4,5]. AIN can lead to acute renal failure in hospitalized patients, and those who experience AKI are at increased risk of developing chronic kidney disease in the future [6-8]. Physicians should be cautious when prescribing AMX, as drug-induced kidney injury, although rare, can occur. We present the case of a 56-year-old woman who developed AKI and HUS after receiving AMX for a tooth infection.

## Case presentation

A 56-year-old woman presented to the emergency department with nausea, vomiting, and diarrhea lasting for four days. Her past medical history included gastroesophageal reflux disease and recent AMX use for a tooth infection, with the last dose administered approximately one day before her hospital arrival. Physical examination revealed mild generalized abdominal tenderness, decreased skin turgor, and dry oral mucosa, but no other abnormalities. She was started on intravenous fluids. She reported markedly decreased urine output over the past four days. Her complete blood count is shown in Table 1. The complete metabolic profile is detailed in Table 2. The peripheral smear results are presented in Table 3, and the urinalysis findings are displayed in Table 4.

**Table 1:** Complete blood count results on admission The complete blood count results revealed anemia and significant thrombocytopenia, along with a borderline elevated white blood cell count.

Parameter	Values	Reference range
Hemoglobin	9.1 g/dl	11-18 g/dl
White blood cells	11.34 × 10^3^	4,500 and 11,000 per μl
Mean corpuscular volume	83.8 fL	80-99 fL
Platelet	17,000/uL	150,000-450,000/uL
Red blood cells	3.6 × 1,012/L	3.5-5.5 × 1,012/L
Hematocrit	25.80%	36-46%

**Table 2:** Comprehensive metabolic panel results on admission The metabolic panel revealed severe renal impairment and a borderline elevated white blood cell count.

Parameter	Value	Reference range
Blood urea nitrogen	110	7-25 (mg/dl)
Creatinine	9.23	0.70-1.33 (mg/dl)
eGFR	4	>OR = 60 (mL/min/1.73 m^2^)
Sodium	130	135-146 (mmol/L)
Potassium	3.7	3.5-5.3 (mmol/L)
Chloride	104	98-110 (mmol/L)
Carbon dioxide	30	20-32 (mmol/L)
Calcium	9.4	8.6-10.3 (mg/dL)
Bilirubin total	2	1.2 mg/dL

**Table 3:** Peripheral blood smear results for red cell morphologies on admission The peripheral blood smear revealed schistocytes, acanthocytes, and burr cells, all graded as +1 (few), +2 (moderate amount), and +3 (numerous/marked amount). These findings are compatible with TMA. TMA, thrombotic microangiopathy

Red cell morphology	Grade
Poikilocytosis	2+
Acanthocytes	2+
Anisocytes	1+
Burr cell	1+
Schistocytes	1+
Microcytes	1+

**Table 4:** Urinalysis results on admission The urinalysis revealed alkaline urine and proteinuria.

Parameter	Value	Reference range
Color	Yellow	Yellow
Appearance	Cloudy	Clear
Bilirubin	Negative	Negative
Ketones	Negative	Negative
Specific gravity	1.014	1.001-1.035
Occult blood	Negative	Negative
PH	7.5	5.0-8.0
Protein	positive	Negative
Nitrite	Negative	Negative
Leukocyte esterase	Negative	Negative
Glucose	Negative	Negative

Her creatinine level was 10 mg/dL, the spot urine protein-to-creatinine ratio was 4.6 g/L, and her serum albumin was 3.6 g/dL. AMX was discontinued immediately, and both HUS and thrombotic thrombocytopenic purpura were suspected. Her ADAMTS13 activity level was within the normal range. Shiga toxin testing and stool culture were negative. Plasmapheresis was arranged due to the suspicion of HUS. Serologies, including ANA, P-ANCA, C-ANCA, and complement levels, were normal. Hemodialysis was initiated due to poor urine output and persistently elevated serum creatinine, despite adequate fluid resuscitation.

**Figure 1:**
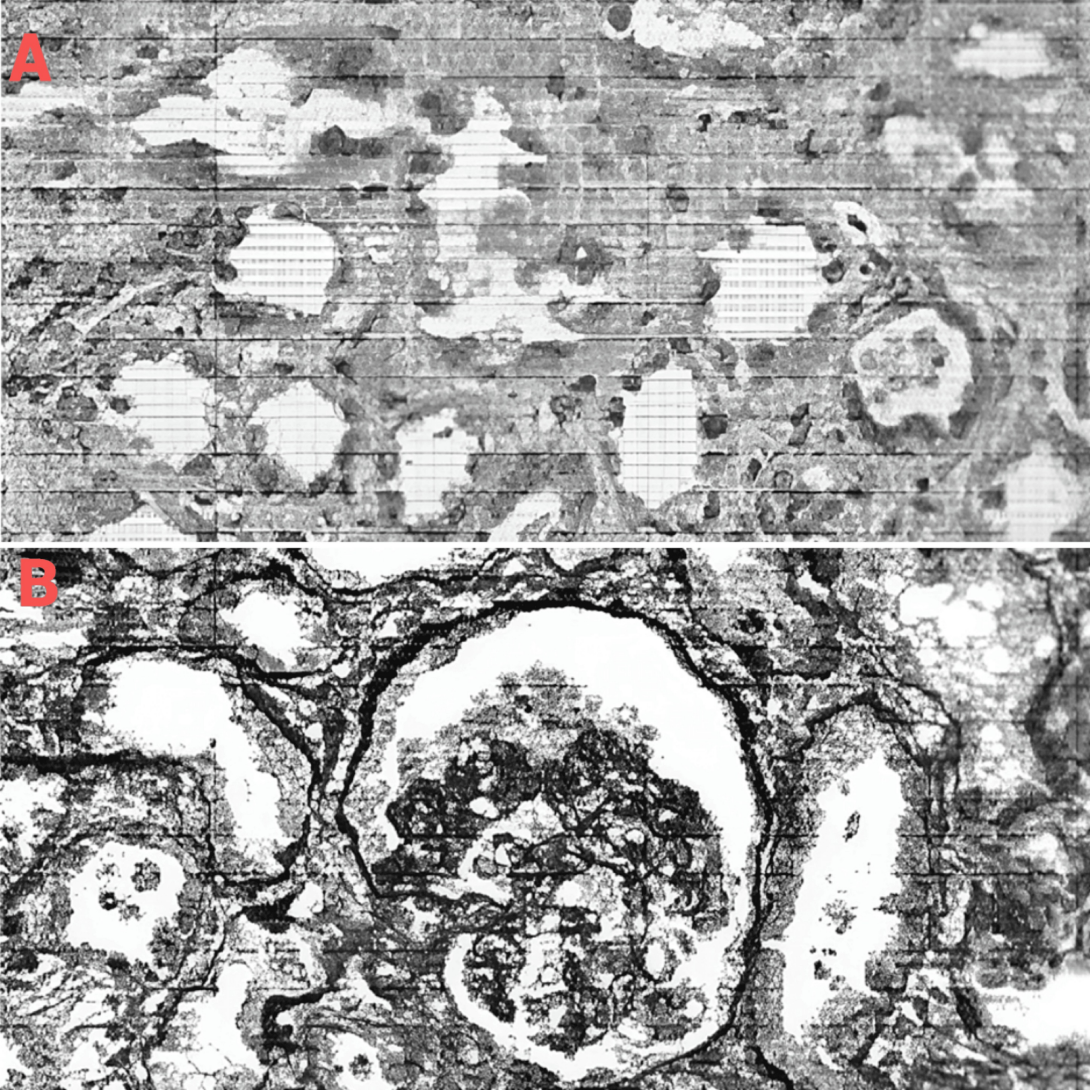
Kidney biopsy (A) Acute tubular necrosis is depicted, showing the sloughing of tubular cells responsible for granular cast formation. (B) Focal segmental glomerulosclerosis with collapsing features is shown. This finding was a less prominent aspect of the biopsy.

The kidney biopsy revealed a unique histological picture of glomerular damage consistent with acute tubular injury and focal segmental glomerulonephritis. The findings included patchy moderate interstitial edema, sparse cortical inflammation with mononuclear leukocytes, rare neutrophils and eosinophils, and scattered granular red-brown casts in distal tubular lumina. The biopsy results are shown in Figure 1A and Figure 1B.

A presumed diagnosis of drug-induced kidney injury with concurrent TMA was made. She was discharged on the 10th day, when her creatinine level had decreased to 3.48 mg/dL. She attended a nephrology clinic for follow-ups and was scheduled for weekly hemodialysis. Her renal function has since recovered.

## Discussion

HUS is a TMA characterized by thrombocytopenia, hemolytic anemia, and renal failure [9]. Our patient presented with low platelet counts, hemolytic anemia (evidenced by schistocytes on peripheral smears), and renal failure. Both hemodialysis and peritoneal dialysis are effective treatments for HUS [10]. In this case, the patient was treated with plasmapheresis and hemodialysis.

AMX has been reported to cause renal damage, with literature supporting its role in renal failure pathogenesis [9]. AICN is the most commonly described renal adverse effect associated with this antibiotic. Sjövall et al. noted profound crystalluria related to AMX, emphasizing that renal clearance of the drug is not dependent on its plasma concentration [11].

Our patient developed AKI following AMX treatment for a tooth infection, with all other causes of AKI ruled out. The renal biopsy confirmed the tubular injury. While renal impairment is typically reversible upon discontinuation of the offending agent, our patient required temporary hemodialysis before her kidney function normalized. Identifying the cause of HUS is crucial, as early diagnosis significantly influences treatment and prognosis.

## Conclusions

Patients who experience a significant rise in creatinine following the administration of β-lactam antibiotics should be evaluated for drug-induced kidney injury and HUS. A kidney biopsy is crucial for confirming the diagnosis. Physicians should be aware of risk factors when prescribing antibiotics, such as advanced age, concomitant use of nephrotoxic drugs, and high dosage. Renal impairment is generally reversible once the offending agent is discontinued. A comprehensive medical history, thorough physical examination, and prompt therapeutic intervention can significantly impact treatment outcomes and prognosis. Large case-control studies are needed to assess risk factors associated with antibiotic-induced HUS and AKI. Therapeutic drug monitoring is essential for individuals at risk of kidney damage.
